# Exploiting Task Constraints for Self-Calibrated Brain-Machine Interface Control Using Error-Related Potentials

**DOI:** 10.1371/journal.pone.0131491

**Published:** 2015-07-01

**Authors:** Iñaki Iturrate, Jonathan Grizou, Jason Omedes, Pierre-Yves Oudeyer, Manuel Lopes, Luis Montesano

**Affiliations:** 1 Chair in Brain-Machine Interface (CNBI) and Center for Neuroprosthetics (CNP), École Polytechnique Fédérale de Lausanne (EPFL), Lausanne, Switzerland; 2 Flowers Team, a joint Inria—Ensta ParisTech lab, Bourdeaux, France; 3 Instituto de Investigación en Ingeniería de Sistemas (I3A), Universidad de Zaragoza, Zaragoza, Spain; Duke University, UNITED STATES

## Abstract

This paper presents a new approach for self-calibration BCI for reaching tasks using error-related potentials. The proposed method exploits task constraints to simultaneously calibrate the decoder and control the device, by using a robust likelihood function and an ad-hoc planner to cope with the large uncertainty resulting from the unknown task and decoder. The method has been evaluated in closed-loop online experiments with 8 users using a previously proposed BCI protocol for reaching tasks over a grid. The results show that it is possible to have a usable BCI control from the beginning of the experiment without any prior calibration. Furthermore, comparisons with simulations and previous results obtained using standard calibration hint that both the quality of recorded signals and the performance of the system were comparable to those obtained with a standard calibration approach.

## 1 Introduction

The field of brain-computer interfaces (BCI) has witnessed largely successful applications in different contexts such as the control of assistive devices or communication restoration [1]. However, their impact has been limited to in-lab applications due to intrinsic limitations of the technology. The brain signals used in BCI have a high variability caused by factors such as non-stationarities [2], task-dependent variations [3], and large subject specificity [4]. To solve these problems, most applications rely on an initial calibration phase that needs to be frequently repeated due to the changing nature of the brain signals. This calibration process is a tedious and time consuming part in most BCI applications that hinders their deployment. First, it impedes an out-of-the-box use of the BCI both in assistive or leisure scenarios. Second, it increases the cost in professional and medical applications such as those carried out in hospitals. Third, calibration is an overload that affects the usability of the system in the long term and, therefore, the introduction and acceptance of such systems.

Previous works have aimed at minimizing and/or simplifying the calibration phase. For instance, in motor imagery [1], the supervised and unsupervised adaptation of classification parameters has shown to avoid inter-session recalibration [5] and to increase the BCI performance [6]. Other approaches have tried to limit the calibration time by using clustering methods in the feature space [7]; ensembles of classifiers built from very large datasets [8]; inter-subject generalization by using a pool of subjects to initialize the classifier [9–11]; or even by generating artificial training datasets via resampling [12].

Despite their successful results, these methods aim at reducing the calibration phase rather than completely removing it. On the other hand, free-calibration BCIs (i.e. *plug-and-play BCIs*) remain scarce as they need to achieve BCI control together with a real-time, transparent, and unsupervised calibration executed in parallel. For these approaches, the main idea is to exploit redundancy or task constraints to limit the possible space of brain signals decoders to a tractable one. In non-invasive BCI, Kindermans et al. have thoroughly shown that such kind of BCI is feasible for P300 spellers [13, 14]. In this approach, the self-calibration speller exploits the multiple repetitions of P300 stimuli and context information of the task, namely word constraints and grammar rules. Similarly, Orsborn et al. have also achieved control from scratch for reaching tasks in an invasive brain-machine interface [15, 16]. They initialized the decoder to a random behavior and updated it during online control using the assumption that the targets should only be reached following a straight line.

This paper presents a new approach and an online study of a self-calibration method for BCI control applications based on error potentials [17, 18]. The key insight of this protocol is that the task to be solved provides constraints that can be exploited to simultaneously calibrate, in an unsupervised fashion, the BCI while controlling the device. With respect to our preliminary works, the current study proposes an alternative algorithm that does not suffer from the limitations of [19], namely failures due to overconfident estimates. Furthermore, it shows the applicability to online scenarios of the planning techniques described and evaluated in [20]. The method has been evaluated online in a reaching task over a discrete grid. Results of eight subjects show that the performance of the system was above that obtained using a standard BCI calibration method in terms of time required to reach the intended goal and in the number of goals achieved for a fixed amount of time. Furthermore, after a certain time, the system is indeed calibrated without incurring any overhead in the long term.

## 2 Methods

### 2.1 Experimental Protocol

**Fig 1:**
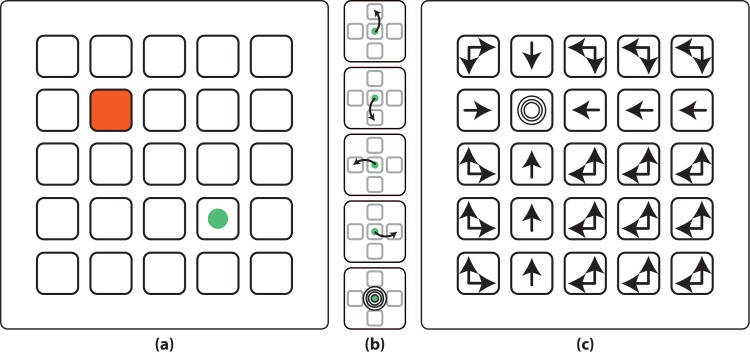
Experimental protocol. (a) Experimental protocol. The protocol showed a 5 × 5 grid with a virtual cursor (green circle) and a goal location (shadowed in red). (b) The cursor could perform five different actions (from top to bottom, move one position up, down, left, right, or performing a goal-reached action). (c) Correct actions (i.e. optimal policy) from each state for the goal exemplified in (a). Extracted from [18] with permission.

The usability of the self-calibration approach was tested under an experimental protocol demonstrated in a prior work [18]. The protocol consisted of a 5 × 5 squares grid, a virtual cursor, and a goal location (see Fig 1). The cursor performs five different instantaneous actions: move one position up, down, left, right; and a goal-reached action, represented as concentric circumferences (see Fig 1b). The time between two actions (inter-action interval) was random within the range [3,3.5] s. The role of the subjects was to assess the cursor actions as correct when the cursor performed (*i*) a movement towards the goal position, or (*ii*) a goal-reached action over the goal position; or as incorrect otherwise (see Fig 1c). These assessments generated the associated evoked potentials for both correct and error conditions (i.e., error-related potentials, ErrP [21]). Note that the goal position was known by the user, but it was unknown by the device. The users were instructed not to move their eyes during the cursor actions, and to restrain blinks only to the resting periods. The experiment duration was fixed to 500 actions performed by the device, with rest intervals every 10 actions. Every time the device reached a target, the target was changed to a different position, with the sequence of target positions the same for all the subjects. The total length of the experiment was around 50 minutes. For more details about the protocol, please refer to [18].

Eight subjects (mean age 28±1.55 years) participated in the experiments after the protocol was approved by the Institutional Review Board of the University of Zaragoza. All participants were asked to read and sign an informed consent form to participate in the study. The participants were seated on a comfortable chair one meter away from a computer screen that displayed all the information related to the experiment. For the executed experiments we used an unsupervised self-calibration approach (c.f. section 2.5) to simultaneously train the error-related potentials (ErrP) [21] classifier and solve the task (i.e., reach the target position). The data recorded during the experiments is publicly available, and can be downloaded from https://github.com/flowersteam/self_calibration_BCI_plosOne_2015.

### 2.2 Data recording and feature extraction

EEG signals were recorded from 16 fronto-central active electrodes using a gTec system, with the ground on AFz and the reference on the left earlobe. EEG data were digitized with a sampling frequency of 256 Hz, power-line notch filtered, and band-pass filtered at [1, 10] Hz. The data acquisition and online processing was developed under a self-made BCI platform. In this work, ErrP features were extracted from channels Fz, FCz and Cz within a time window of [200, 800] ms downsampled by a factor of 8 (being 0 ms the action onset), forming a vector of 57 features [17].

### 2.3 Supervised decoding of error potentials

Before describing the self-calibration method for error-based control, we briefly describe here the supervised approach [17, 18] which will be later extended to an unsupervised setting. The decoding of the EEG signals is done using a Gaussian classifier. The signals are modeled using an independent multivariate normal distribution for each class (i.e. correct denoted as *c* or error as *w*) [4, 22], 𝓝(*μ*_*c*_, Σ_*c*_), 𝓝(*μ*_*w*_, Σ_*w*_). *θ*_*k*_ denotes the set of parameters of each class {*μ*_*k*_, Σ_*k*_} where *k* ∈ {*c*, *w*}. The covariance matrices Σ_*c*_ and Σ_*w*_ are shrinked (*λ* = 0.5) to avoid overfitting when using a large number of features compared to the training number of samples [23].

The probability of a label given a signal is obtained using the Bayes’ rule:

$$\begin{array}{ccc} p(l=j|e,\theta_{j}) & = & \frac{p\left(e|l=j,\theta_{j}\right)p\left(l=j\right)}{\sum_{k=c,w}p\left(e|l=k,\theta_{k}\right)p\left(l=k\right)} \end{array}$$ (1)

where *e* represents the features extracted from the brain signal, *j* ∈ {*c*, *w*} is one of the two classes to be decoded. As we do not have a priori knowledge on the user intended assessment of the action, we assume all labels are equi-probables, i.e. ∀*k*, *p*(*l* = *k*) = 0.5. In this supervised setting, an estimation of the parameters $\hat{\theta }$ is obtained using a set of pairs of signals and labels prior to the actual use of the brain signals.

### 2.4 Error-potential based control

Once trained, the decoder of the previous section can be used to control a device based on the user’s assessment of the behavior of the device [18]. The control task is modeled as a Markov decision process (MDP). An MDP is defined by a triplet (*s*, *a*, *r*) where *s* ∈ *S* and *a* ∈ *A* represent the states and actions, and *r* represents the reward. Actions modify the state of the system according to the corresponding transition model *p*(*s*′∣*s*, *a*). In our grid world protocol, the state represents the cell in the grid and *p*(*s*′∣*s*, *a*) is simply a deterministic transition up, down, left, right, or performing a goal-reached action from the current state *s* according to the action *a*. The reward function encodes the task, in our case it assigns positive reward to the goal state and zero reward to the others. Given this reward function, it is possible to compute the optimal policy (i.e. a function that gives you the action to execute at each state) that maximizes the accumulated reward.

Under the assumption that the user follows optimal policies, the ErrP detector can be used to identify the user intended task *ξ* = *t* among a finite set of tasks *t* = 1, …, *T*. Note that this assumption does not contradict the fact that we also assume a non-informative a priori for the labels, as the latter will vary among optimal policies and will depend on the planner.

The rationale of the algorithm is the following: the actions of the device that agree with the optimal policy will not generate error responses, while those that do not follow the policy will. This can be implemented as a recursive filter to identify the task based on the signals *e* obtained when executing action *a* in state *s*:

$$\begin{array}{ccc} p(\xi =t|e,s,a,\hat{\theta }) & \propto & p\left(e|s,a,\hat{\theta },\xi =t\right)p\left(\xi =t\right) \end{array}$$ (2)

where $p(e|s,a,\hat{\theta },\xi =t)$ needs to take into account the probability of each possible class *k* given the target *ξ* = *t*, the current state *s* and the action *a* executed by the machine:

$$\begin{array}{c} p\left(e|s,a,\hat{\theta },\xi =t\right)=\sum_{k=c,w}p\left(e|l=k,\hat{\theta }\right)p\left(l=k|s,a,\xi =t\right) \end{array}$$ (3)

The previous estimate simply assigns a higher likelihood to those tasks with whom the brain signals are more coherent. Fig 1c shows the optimal policy for a given task, that is, the actions that should not generate error potentials if this is the user’s intended task. The posterior distribution is then used to control the device to the most likely goal using a planning algorithm or greedy heuristic (see [18] for further details).

### 2.5 Self-calibration control

This section presents a self-calibrated algorithm that eliminates the need of calibration prior to the control task. This is a self-calibration approach since it estimates *θ* in an unsupervised manner during operation. Self-calibration control can be achieved by exploiting the structure of the task space, namely the labels that the optimal policy of each task assigns to each state-action pair. These learned labels can be used to train a decoder for each possible task. The remaining question is how to select among the different tasks. For this purpose, we define the following pseudo-likelihood function [20]:

$$\begin{array}{c} P\left(D_{M}|\xi =t\right)\approx \prod_{i=1}^{M}p\left(e_{i}|s_{i},a_{i},\xi =t,D_{-i}\right) \end{array}$$ (4)

$$\begin{array}{c} =\prod_{i=1}^{M}\sum_{k=w,c}p\left(e_{i}|l=k,\xi =t,D_{-i}\right)p\left(l=k|s_{i},a_{i},\xi =t\right). \end{array}$$ (5)

where *D*_*M*_ represents the history of triplets {*a*_*i*_, *s*_*i*_, *e*_*i*_} up to time *M*. The pseudo-likelihood is built using a leave-one-out cross-validation strategy to evaluate the likelihood *p*(*e*_*i*_∣*s*_*i*_, *a*_*i*_, *ξ* = *t*, *D*_−*i*_) of each signal *e*_*i*_ based on a model learned with all the other signals and corresponding actions and states denoted by *D*_−*i*_. For a given target hypothesis *ξ* = *t*, the labels associated to the signals in *D*_−*i*_ can be estimated (i.e. *p*(*l* = *k*∣*s*_*i*_, *a*_*i*_, *ξ* = *t*)), enabling the training of a target-specific classifier for each task *ξ* = *t*. In a nutshell, Eq 5 assigns higher likelihood to tasks in which their optimal policies match the labels predicted with the model learned with *D*_−*i*_. As detailed in [20], the underlying assumption is that the subject is coherent throughout the experiment, providing similar signals for the same set of labels.

The pseudo-likelihood is computed from two terms. *p*(*l* = *k*∣*s*_*i*_, *a*_*i*_, *ξ* = *t*) represents the probability distributions of the labels according to a task, the executed action, and the current state. This term only depends on what action is correct at each state according to task *ξ* = *t*.

The other term, *p*(*e*_*i*_∣*l* = *k*, *ξ* = *t*, *D*_−*i*_), represents the likelihood of the signal given the label provided by the task and the previous history. In principle, one could use directly the same likelihood model as in Eq 3, with *θ* estimated from *D*_−*i*_. However, such a model results in very fast convergence and a high sensitivity to outliers (e.g. incorrect assessments by the user). In order to improve the robustness of the method, we propose to marginalize out *θ* using a non-informative (Jeffrey’s) prior (see [24], page 88). The resulting likelihood function is a t-student distribution with heavier tails:

$$\begin{array}{ccc} p(e|l=k,\xi =t,D_{-i}) & = & t_{n-d}(e|\mu_{k},\frac{\Sigma_{k}\left(n+1\right)}{n(n-d)}) \end{array}$$ (6)

where *μ*_*k*_ and Σ_*k*_ are the corresponding empirical mean and covariance matrix, *n* is the number of signals, and *d* is the dimensionality of a signal feature vector.

As in the calibrated case, the device can use the task estimates to select the next action greedily according to the corresponding optimal policy [18]. However, this strategy is prone to fail during the initial stages due to the large uncertainty introduced by the lack of calibration. To alleviate this difficulty, we incorporate this uncertainty in the decision making and use a look-ahead planning based on [20].

Unlike the calibrated case, we do not only have uncertainty on the tasks but also on the signal models. Indeed, each task is associated with a different set of signal-label pairs leading to a different classifier. Hence our agent must collect data to discriminate between tasks but also to understand the structure of the signal in the feature space. These two sources of uncertainty can be unified as a measure of uncertainty on the signal space, as described in [19], which can be approximated on the label space through a sampling procedure [20].

The agent will then target areas of higher uncertainty, to reduce it by collecting more evidence in these areas. To do so, the uncertainty is computed for each state-action pair and used as a reward function to be maximized by the agent (using value iteration [25]). The system follows the optimal policy for this reward function and switches to a pure exploitation of the task, ignoring the uncertainty, after reaching the desired confidence level (see section 2.6.2).

### 2.6 Practicalities

We now describe some implementation details that were used in the experimental evaluation of the method. The code is available under a GNU GPL license and can be found at https://github.com/jgrizou/lfui.

#### 2.6.1 Power information

One critical difficulty of learning from unlabeled signals is for cases where the signals can be interpreted in a symmetric way. In other words, it is possible to obtain the same likelihood by switching the labels. Such misinterpretation is possible in environments with symmetric properties (see [20] for reaching tasks and [13, 14] for a speller). For the domain of our experiments the symmetries can be disambiguated using the goal reached action, but, in general, symmetric tasks (e.g. opposite corners) will be hard to discriminate and might produce an inconsistent agent behavior.

Prior information about the differences in power between correct and error signals can be used to increase the robustness of the method—helping to remove world symmetries (e.g. equal likelihood for models with symmetric labels). In single trial classification power spectral information results in lower performance than temporal features, but their combination produces more robust detectors [26]. However, in our case it is possible to exploit the fact that EEG signals associated to the error class have on average higher power than the ones associated to the correct class.

This information is added to the pseudo-likelihood model of Eq 5 as follows. The average power information of each class is computed as the weighted mean of the signals’ power. The weights are the probabilities of the label given the signal and the class parameters

$$\begin{array}{c} p_{k}\left(\xi =t\right)=\frac{\sum_{i=1}^{M}p\left(l=k|e_{i},\theta_{k}\right)\;\;e_{i}^{T}e_{i}}{\sum_{i=1}^{M}p\left(l=k|e_{i},\theta_{k}\right)}. \end{array}$$ (7)

with *θ*_*k*_ the corresponding parameters of each class *k*, and the power computed as the inner product of *e*_*i*_. The pseudo-likelihood is multiplied by the ratio of the power associated to the error class over the power of the correct class:

$$\begin{array}{c} \bar{P}\left(D_{M}|\xi =t\right)=P\left(D_{M}|\xi =t\right)\frac{p_{w}\left(\xi =t\right)}{p_{c}\left(\xi =t\right)}, \end{array}$$ (8)

which assigns, given two symmetric labeling, higher probability to the one where the average power of error signals is larger.

#### 2.6.2 Identifying the goal

A practical issue when running this algorithm is to decide when the task has been successfully identified. This decision has to take into account the uncertainty about the task. We use a thresholding method and define as *W* ^*t*^ the minimum of pairwise normalized likelihood between tasks *ξ* = *t* and all other tasks:

$$\begin{array}{c} W^{t}=\min_{x\;\in \;1,\ldots ,T\setminus \{t\}}\frac{P(D_{M}|\theta ,\xi =t)}{P\left(D_{M}|\theta ,\xi =t\right)+P\left(D_{M}|\theta ,\xi =x\right)} \end{array}$$ (9)

When it exists a task *ξ* = *t* such that *W* ^*t*^ exceeds a threshold *β* ∈ [0.5, 1] we consider that it is the one the subject has in mind.

Once a task is identified with confidence, the device switches to a greedy policy following the optimal one to reach the estimated goal. Once there, it starts a new task. At this point the device can use the ErrP signals of the previous task as prior information. Indeed, it assigns to the signals the labels of the optimal policy of the identified task. Note that this prior information is used by the device for all the subsequent error potentials and it results on faster task identification since the classifier parameters are better estimated from the beginning [20]. In fact, when tasks are correctly identified, the resulting classifier is equivalent to a calibrated one trained with a similar number of ErrP signals.

### 2.7 Self-calibration control analysis

Firstly, the recorded potentials during the online experiment were studied to assess whether their characterization was similar to those obtained in previous works. Notice that, contrary to classical calibration approaches where the error ratio remained fixed [17, 18], the error ratio in our approach could not be fixed due to the exploration algorithm and the unsupervised nature of the problem. For this analysis, signals for each condition (error and correct movements performed by the device) were grand averaged for the time window [−200, 1000] ms, where 0 ms represents the action onset.

Secondly, the self-calibration online task was analyzed with the following metrics: number of steps to reach the first target; total number of (incorrect or correct) targets reached during the whole experiment (500 steps) and number of correct and incorrect targets reached. To evaluate the learning progress during the first target, the instantaneous error rate was computed as a moving average over the last 10 actions. Finally, we compared the quality of the labels acquired by the self-calibration approach with the one that would be obtained using a supervised calibration method. Note that, when the device reaches an incorrect target, some signals are incorrectly labeled (those corresponding to states whose optimal action differ from the intended target). To this end, we computed the percentage of labels estimated correctly by the self-calibration approach and the ten-fold accuracy obtained using these labels, and compared it to the ten-fold accuracy obtained with ground-truth labels.

Thirdly, the proposed approach was compared with standard calibration procedures. In order to perform this comparison, the important factors to analyze are the quality of the signals acquired, and the labels quality learned from the self-calibration procedure. In principle, if both the signals acquired and labels learned are equivalent to those of a standard calibration, the performance of the system should also be equivalent. To this end, we took advantage of the overlap of three subjects between the current work and our previous work where the protocol was the same but following a classical supervised calibration [18]. For this analysis, we compared first the quality of the EEG signals in terms of grand averages (error minus correct grand averages); as well as the accuracies obtained during the online control phase following the standard calibration versus the accuracy obtained during the self-calibration session.

Finally, we performed an offline analysis to compare the self-calibration online results with a standard calibration using the same signals acquired during the online experiment. The number of calibration trials required for each subject was estimated by splitting the dataset into a training set formed by 300 trials, and a test set composed of the remaining ones (200). Then, a classifier was trained using an incremental number of trials from the training set and evaluated on the test set. This process was repeated ten times shuffling the order of the trials. The number of trials required for calibration was selected as the one for which the accuracy reached a plateau for at least five consecutive trials. Once a classifier was trained using the computed number of trials, the ErrP-based control algorithm (see subsection 2.4) was run using the remaining data available. These simulations were run 100 times for each subject to extract the task metrics comparable to those obtained in the self-calibration approach (number of steps needed to reach first target and number of reached targets).

## 3 Results

### 3.1 EEG results

**Fig 2:**
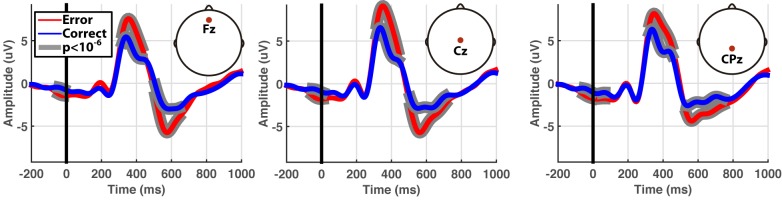
Grand-averaged signals for Fz, FCz and CPz channels. Red and blue lines represent averaged signals for the error and correct conditions respectively, where the movement onset is marked as time 0 ms. Statistical differences (unpaired t-tests, p-values < 1·10^−6^) between error and correct averages are marked by shadowed areas.

Fig 2 shows the grand-averaged signals obtained during the online experiments. Error and correct assessments generated different evoked responses posterior to the device actions. The difference between error and correct potentials appeared on fronto-central locations at around 400 and 600 ms, where the error potential exhibited significantly larger positive and negative peaks respectively (unpaired t-tests, p < 1·10^−6^). These evoked responses were in line with previous experiments using error potentials [17, 18].

### 3.2 Online self-calibration results

Table 1, left shows the results obtained during the online experiments. On average, users were able to reach 6.88 correct targets in the 500 steps of the experiment. However, users also reached an average of 1.50 incorrect targets, significantly greater than zero (one-sample t-test, *t*_7_ = 3.55, *p* = 0.009). Whereas users needed 165 steps on average to reach the first target, subsequent targets were reached after 60 ± 24 steps on average across subjects. Note that the first target needed a substantially larger amount of data to converge due to the uncertainty not only on the target to reach, but also on the decoder of brain signals.

**Table 1:** Task results.

Online self-calibration					Simulated standard calibration			
Subject	Calib. trials	Steps 1^*st*^	#Correct	#Incorrect	Calib. trials	Steps 1^*st*^	#Correct	#Incorrect
**S1**	0	101	16	2	213	239.71 ± 9.2	10.33 ± 1.48	0.14 ± 0.38
**S2**	0	131	9	1	246	286.73 ± 14.75	5.50 ± 0.93	0.03 ± 0.22
**S3**	0	265	3	1	190	323.67 ± 56.32	1.78 ± 0.76	0.01 ± 0.10
**S4**	0	232	2	2	101	336.98 ± 75.50	0.59 ± 0.71	0.10 ± 0.33
**S5**	0	132	5	3	234	317.68 ± 24.85	2.60 ± 0.83	0.02 ± 0.14
**S6**	0	142	10	0	300	340.31 ± 15.08	4.62 ± 0.81	0.09 ± 0.29
**S7**	0	79	4	3	82	310.24 ± 103.64	0.48 ± 0.82	0.36 ± 0.66
**S8**	0	240	6	0	252	290.47 ± 12.7	5.91 ± 0.70	0.05 ± 0.22
**mean**	**0.00**	**165.25**	**6.88**	**1.50**	**202.25**	**305.72 ± 32.97**	**3.97 ± 3.32**	**0.10 ± 0.11**

**Fig 3:**
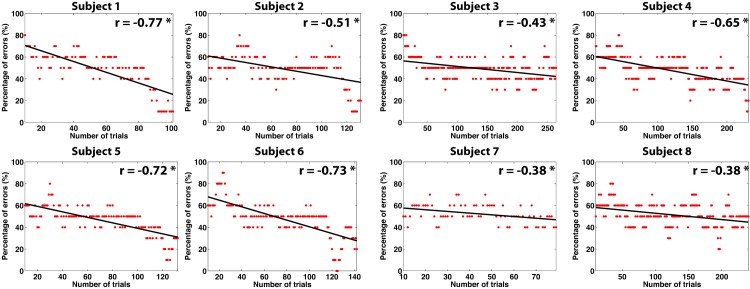
Percentage of errors performed by the device during the last 10 trials, as a function of the number of trials (only during the first target). Additionally, the tendency line and the correlation value are also shown.

**Fig 4:**
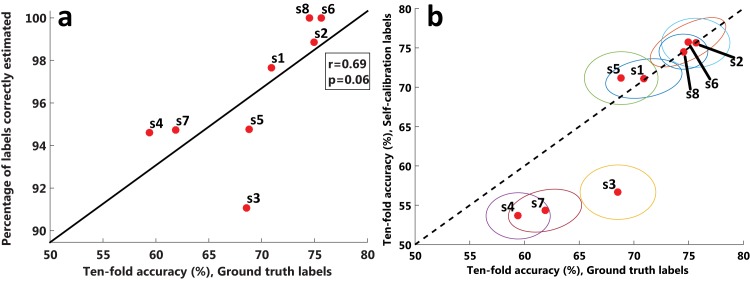
Learned labels quality. (a) Ten-fold accuracy using the ground-truth labels (x-axis) vs percentage of the labels correctly learned by the self-calibration protocol (y-axis), together with the tendency line, where each red dot represents one subject. (b) Ten-fold accuracy using the ground-truth labels (x-axis) compared against the ten-fold accuracy using the labels learned by the self-calibration approach (y-axis). Each dot represents one subject, whereas the ellipsoid represents the uncertainty computed from the ten-fold accuracies. Every dot below (above) the dashed line implies that the self-calibration approach had worse (better) accuracies than the ground-truth labels.

Fig 3 shows the evolution of the ratio of errors within the last ten movements of the device until reaching the first target. The results for each subject show a clear decreasing trend in the percentage of errors significant for all the subjects (*p* < 0.001) with an average correlation of *r* = 0.57±0.17. Expectedly, these results show that the error rate was variable throughout the experiment. Nonetheless, the negative tendency also indicated that the system was able to iteratively learn the task even for the first target using completely unsupervised data. Regarding the labels quality, Fig 4a shows the ten-fold accuracy obtained with the ground truth labels, compared against the percentage of labels correctly learned by the self-calibration protocol. The results show an almost significant correlation between the two variables (*r* = 0.69, *p* = 0.06). Nonetheless, the ratio of labels correctly assigned was always higher than 90%, indicating that even for subjects with low accuracies (< 60%), most of the labels are correctly estimated. Fig 4b shows the ten-fold accuracy obtained with ground truth labels versus the accuracy obtained with the labels estimated from self-calibration. Interestingly, there seemed to be two clusters of subjects: those who were not affected by the labeling quality composed of five subjects with very similar accuracies to the ones obtained with ground truth labels, even when not all the labels were correctly assigned; and other subjects where the labeling of the self-calibration approach affected the separability of the data acquired with a decrease of 8.41% of accuracy on average.

### 3.3 Comparison with standard calibration

**Fig 5:**
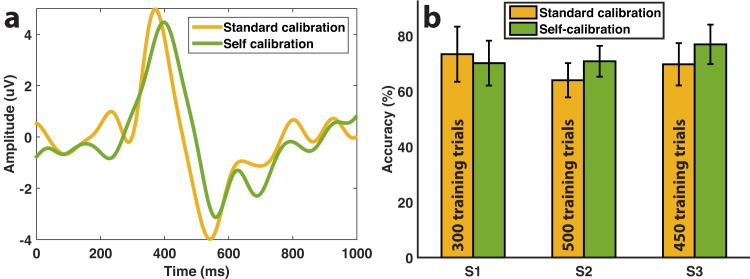
Signal and accuracy comparison with standard calibration. (a) Difference average (error minus correct grand averages) at channel FCz for the 3 subjects that performed the standard calibration and self-calibration protocols. (b) Online mean classification accuracy (± std) of the three subjects after following each calibration procedure, together with the number of calibration trials used for training in the standard calibration approach.

Fig 5 shows the comparison with the results obtained in our previous work (see [18]) in terms of EEG signal and classification performance, based on the subjects that followed both calibration approaches (standard calibration and self-calibration). Regarding the grand-averaged signals (Fig 5a), no substantial differences were found on the difference potential, with only slight variations in the amplitude of the peaks. Despite small variations of around 20 ms were found in the peak latencies, these values are below our current event synchronization resolution of 62.50 ms, and thus were mainly due to noise.

Similarly, the accuracies obtained during the online task (Fig 5b) following the two calibrations was significantly similar (Bonferroni-corrected unpaired t-test, *p* > 0.1), demonstrating that the self-calibration approach did not result in a decrease in the classification performance. Furthermore, the performance of the self-calibration was slightly higher than that of the standard calibration, probably due to a learning effect on the user side. Fig 5b also shows the number of calibration trials needed for each subject, an average of 417 trials. In the same number of trials, the zero-calibration approach allow to reach an average of 8±1 targets, and thus perform the reaching task much more efficiently.

**Fig 6:**
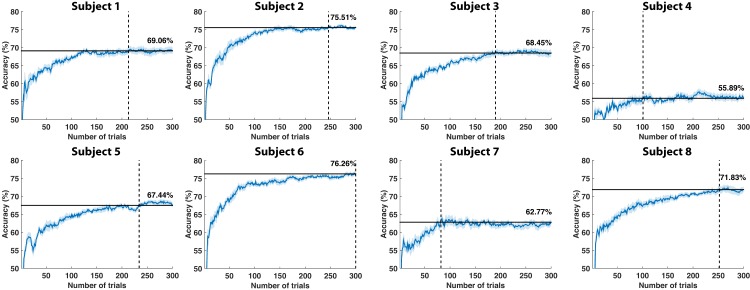
Accuracy from simulated standard calibration. Simulated calibration represented as accuracies computed by increasing the number of trials of the training dataset (x-axis) and testing the classifier with a fixed test set of 200 trials. In order to have a confidence measure (shadowed areas), this procedure was repeated 10 times while shuffling the data. The horizontal solid line represents the accuracy plateau (also shown above the line), whereas the dashed vertical line shows the number of trials needed to reach the plateau.

Regarding the simulation results, Fig 6 shows the simulated training accuracies for each subject. As can be seen, the accuracies varied substantially from subject to subject, with a mean performance of 68.40%±6.69. Nonetheless, the accuracy reached a plateau for all of them, where the mean number of calibration trials to reach this point was of 202±75. This analysis served to run the simulations of an ErrP-based control using a supervised calibration (see Table 1, right). In this case, the number of correctly reached targets was significantly worse than the self-calibration approach (paired t-test, *t*_7_ = −3.67, *p* = 0.008). The number of steps to reach the first target was also significantly worse than the steps following self-calibration (*t*_7_ = −6.08, *p* = 0.001). Notice that for the standard calibration the number of steps to reach the first target include those steps used for supervised calibration. We can further compare the number of steps during self-calibration with the number of calibration trials. In principle, it would seem reasonable that, for those subjects needing more calibration trials, the self-calibration approach might need more trials to reach the first target. However, this hypothesis was falsified and no correlation was found (*r* = 0.02, *p* = 0.97).

## 4 Discussion

This paper presented an online study where eight subjects controlled a cursor using EEG error-related potentials with a self calibration of the decoder. All the subjects performed an online experiment, where the task (i.e. reaching a target position) and the classification parameters were learned in parallel, in an unsupervised way and transparent to the user. The results showed how both task and classifier could be learned, outperforming standard calibration times in terms of whole execution time of the experiment.

The calibration phase is indeed one of the main limitations of current BCI approaches, as they strongly limit the usability of these systems and their applications in out-of-the-lab conditions [1]. Despite recent approaches having demonstrated that it is possible to greatly reduce the calibration time, only a few works have succeeded in removing this phase. As stated in the introduction, Kindermans et al. exploited the multiple repetitions of P300 stimuli and context information of the task to have a zero-calibration P300 approach [13, 14]. Specifically, they exploited different particularities of a P300 speller: first, that among the multiple stimulations, only one event out of six encoded a P300 potential in the speller paradigm (i.e. a fixed P300 ratio); second, the multiple repetitions needed to increase the signal-to-noise ratio of the signal; and third, the ability of rolling back and re-learning classifiers thanks to language models. Indeed, in the P300 speller problem only one row and one column should elicit a P300 response when flashed. Compared with their approach, we could not exploit these constraints, and thus our only constraint was that of having a finite number of reaching positions. As a result, we also needed to add an intelligent planning and exploration algorithm whose objective was to acquire data that maximized the difference between confounded targets (see [19, 20] for details on the exploration algorithms and implementation).

Comparisons with the standard calibration procedure illustrate the advantages of such an adaptive system. First, it is possible to start the operation of the device from the very beginning, allowing to perform the task more efficiently (see Table 1). Second, the obtained accuracies are as good than those obtained with a calibration procedure (see Figs 4 and 5). Therefore, there is not an expected loss of performance in the long term. Third, there is no need for expert supervision or ad-hoc stopping criterion during the self-calibration process (e.g. number of required trials, time or performance constraints) as the system adapts automatically to the user specific behavior.

Notice, however, that an important difference between a standard calibration and self-calibration is the uncontrolled ratio of error vs correct trials. Indeed, it is common in the literature that ErrP calibrations follow fixed ratios between 20% or 30% [17, 18]. Even though the use of ErrP in practical applications cannot assure a stationary percentage of errors, this ratio has empirically shown to generate more distinguishable EEG patterns and better accuracies [17]. As our planning method cannot guarantee the same agent behavior than during a typical calibration phase, the quality of the signals generated by the users might be affected, with strong variations in the error rate from random behaviors (80% of errors) to close to optimal behaviors (below 10%), see Fig 3. Nonetheless, as shown in Fig 5, we did not find any substantial differences neither at the signal level nor at the classification level between standard calibration (with a fixed rate of 30%) and our proposed self-calibration. However, these results have to be taken carefully, as the number of subjects was not enough to draw any definite conclusion and thus further tests will be required.

Although the unsupervised approach attains similar results in terms of accuracy, and insignificantly different grand average signals when compared to those obtained using supervised calibration (see Fig 5), simulations showed differences in performance namely between the number of incorrect targets (see Table 1). There are several plausible explanations for the larger number of wrong targets during self-calibration. First, initial targets do not use a stable classifier and, consequently, it is more probable to misclassify some signals leading to a different target from the intended one. Second, self-calibration also reaches more targets and has more chances to fail. Third, there is no difference between calibration and control phases which may also induce some confusion on the users since the device sometimes aims to reduce its uncertainty on the signals instead of progressing towards the goal. Future work is required to understand the impact of each of these possible reasons and, if possible, to derive a strategy that minimizes wrong targets.

Another influential limitation of the proposed approach is that, with the current algorithm implementation, the users cannot (or should not) switch the desired target during one reaching operation. Under this circumstance, the system could be affected in two ways depending on the relative distance between targets or number of switches among others: first, it could increase the convergence time exponentially; and second, it may severely affect the labeling quality obtained once the system converges to one target. As a possible solution, a target reset function could be implemented by explicitly modeling two possible target locations instead of one. Nonetheless, further work will be needed to understand the impact that this target switches may have on the proposed system and its usability.

An important aspect of the proposed approach is that not only it serves as a calibration system from scratch, but may serve as well as a procedure that can constantly adapt to the user signals reducing the effect of possible non-stationarities. Thus, the proposed approach could serve as a complement rather than an alternative to standard calibration approaches. For instance, the self-calibration algorithm could be run after a standard calibration of few minutes where the user gets accustomed to the protocol and data can be recorded in a supervised manner. In fact, the results showed how several subjects (bottom left subjects in Fig 4b) were affected by the self-calibration, and thus might have benefited from a supervised warm-up period.

An avenue for future research is how to exploit constraints to develop self-calibration BCIs for other tasks or brain signals. On one hand and regarding the control signals, there have been numerous works in the invasive and non-invasive community showing how it is possible to control devices for reaching tasks (e.g. motor rhythms [27], slow-cortical potentials [28] or electrocorticography [29]). We postulate that, as long as there is a finite number of possible targets to reach, our self-calibration approach could be applicable. This idea was partially followed by the invasive community [15, 16], where they studied how to minimize calibration in a center-out reaching task assuming that reaching a target should be done in a straight line. We believe that it would be possible to further extend the algorithm to deal with unknown targets in such scenarios. On the other hand, the use of our proposed self-calibration approach on other BCI tasks solely depends on the existence of task constraints that can be used to model all the possible outcomes of the executed task. These further studies may confirm the usability of the proposed self-calibration approach, improving this way the usability of BCI systems for out-of-the-lab applications.
